# Top Caregiver Concerns in Rett syndrome and related disorders: data from the US Natural History Study

**DOI:** 10.21203/rs.3.rs-2566253/v1

**Published:** 2023-03-20

**Authors:** Jeffrey Lorenz Neul, Timothy A. Benke, Eric D. Marsh, Bernhard Suter, Lori Silveira, Cary Fu, Sarika U. Peters, Alan K. Percy, Rett syndrome Natural History Study Group

**Affiliations:** Vanderbilt University Medical Center; University of Colorado School of Medicine: University of Colorado Anschutz Medical Campus School of Medicine; Childrens Hospital of Philadelphia; Baylor College of Medicine; University of Colorado School of Medicine: University of Colorado Anschutz Medical Campus School of Medicine; Vanderbilt University Medical Center; Vanderbilt University School of Medicine; University of Alabama at Birmingham; Vanderbilt University Medical Center

**Keywords:** Rett syndrome, CDKL5, FOXG1, MECP2 Duplication, Neurodevelopmental disorders, caregiver concerns

## Abstract

**Objective:**

Recent advances in the understanding of neurodevelopmental disorders such as Rett syndrome (RTT) has enabled development of novel therapeutic approaches that are currently undergoing clinical evaluation or are proposed to move into clinical development. Clinical trial success depends on outcome measures that assess clinical features that are most impactful for affected individuals. To determine the top concerns in RTT and RTT-related disorders we asked caregivers to list the top clinical concerns in order to gain information to guide the development and selection of outcome measures for future clinical trials.

**Methods:**

Caregivers of participants enrolled in the US Natural History Study of RTT and related disorders were asked to identify the top 3 concerning problems impacting the affected participant. We generated a weighted list of top caregiver concerns for each of the diagnostic categories and compared results between the disorders. Further, for Classic RTT, caregiver concerns were analyzed by age, clinical severity, and common RTT-causing mutations in *MECP2*.

**Results:**

The top caregiver concerns for Classic RTT were effective communication, seizures, walking/balance issues, lack of hand use, and constipation. The rank order of the frequency of the top caregiver concerns for Classic RTT varied by age, clinical severity, and specific mutations, consistent with known variation in the frequency of clinical features across these domains. The frequency of caregiver concern for seizures, hand use, and spoken language increased in relation to clinician assessed severity in these clinical domains, showing consistency between clinician assessments and caregiver concerns. Comparison across disorders found commonalities in the top caregiver concerns between Classic RTT, Atypical RTT, *MECP2* Duplication Syndrome, CDKL5 Deficiency Disorder, and FOXG1 Syndrome; however, distinct differences in caregiver concerns between these disorders are consistent with the relative prevalence and impact of specific clinical features.

**Conclusion:**

The top caregiver concerns for individuals with RTT and the RTT-related disorders reflect the impact of the primary clinical symptoms of these disorders. This work is critical in the development of meaningful therapies, as optimal therapy should address these concerns. Further, outcome measures to be utilized in clinical trials should assess these clinical issues identified as most concerning by caregivers.

## Introduction

Rett syndrome (RTT) is a severe neurodevelopmental disorder (NDD) that predominantly, but not exclusively [1], affects girls and women and is characterized by regression with loss of acquired spoken language and volitional hand use, disrupted or absent ambulation, and repetitive hand movements [2]. Affected individuals are impacted by a variety of additional clinical problems such as seizures, autonomic and breathing abnormalities, growth failure, scoliosis, and gastrointestinal and nutritional symptoms [3–5]. RTT is caused, in the majority of cases, by loss of function mutations in the X-linked gene *Methyl-CpG-binding Protein 2* (*MECP2*) [6, 7]. Animal models of RTT [8–11] provide insight into underlying pathophysiology and facilitate the development of potential therapeutic interventions with the potential to significantly benefit affected people or even modify the disease course[12]. This has led to the initiation of clinical trials in RTT [13–15], and the proposal of additional trials to evaluate novel treatment approaches.

Critical to successful clinical therapeutic development is detailed knowledge about the disease course, clinical features, and availability of outcome measures that are both psychometrically valid as well as assess critical clinical domains. Extensive information on the spectrum of clinical features and disease progression in RTT has been acquired from the US Natural History Study (NHS) of RTT and Related Disorders, which enrolled people with RTT and disorders with clinical and genetic relationships to RTT: *MECP2* Duplication Syndrome (MDS); CDKL5 Deficiency Disorder (CDD); and FOXG1 Syndrome (FS). This information, combined with other large disease databases [16], has been instrumental in establishing clinical trial readiness through the development of outcome measures [17, 18] and identifying putative biomarkers [19–21]. While these efforts are essential for clinical trials, knowing what clinical issues and problems are most concerning and impactful for affected individuals is necessary to develop therapies that meaningfully address these concerns. Outcome measures are needed that assess those impactful problems relevant to affected individuals and their caregivers [22]. The US Food and Drug Administration (FDA) recognizes the importance of receiving meaningful input from affected individuals on the most important concepts (disease symptoms and impact) to inform the development of outcome measures [23] and has provided guidance on methods to obtain this information from affected people and other key stakeholders [24]. A challenge in severe NDDs such as RTT is that affected people have markedly impaired communication precluding direct ascertainment from the affected individuals. Caregiver reports of meaningful issues and concerns provide a way to develop this understanding and have been utilized in other severe NDDs [25–27]. The FDA has recognized that such caregiver reported information is needed for affected individuals with cognitive limitations [24].

To identify the top caregiver concerns in RTT and RTT-related disorders, we utilized the US NHS data. From 2014–2021, parents or caregivers were asked at every study visit to select the top concerns for the affected individual under their care from a list of potential issues including twenty-one specific concerns and an option to include any other specific issue not listed. Our objective was to identify the top caregiver concerns in Classic RTT and evaluate variation in the top caregiver concerns in relation to age, *MECP2* mutation, overall clinical severity, and specific clinical features such as seizures, hand use, ambulation, and spoken language. Furthermore, we sought to characterize the top caregiver concerns in Atypical RTT, MDS, CDD, and FS, and compare the concerns across the disorders. The work described here provides critical information on the top caregiver concerns in these disorders, identifying similarities and important differences, and represents important information identifying clinical issues that new therapies should target and can help guide the development and selection of outcome measures that assess most meaningful concerns.

## Methods

### Creation of top caregiver concern list

The Rett syndrome and Rett-related Disorders Natural History Study (NHS) evaluated participants from 2006-2021 through three rounds of funding from the NIH (HD061222). In 2014 (NHS #3, NCT02738281), a major revision of the data capture forms was initiated which included asking caregivers to rank the top three concerns for the affected participant at each visit. To capture top caregiver concerns, at each visit the caregiver was asked to identify the top three concerns in order (First Concern, Second Concern, Third Concern). They were provided with a drop-down menu of twenty-one concerns that had been identified from review of the published literature for RTT and discussion amongst expert clinicians to create a list of potential concerns that represented ‘Disease Defining Concepts’ such as impaired hand use, communication difficulties, problems walking, and repetitive hand movements, and other commonly observed clinical features such gastrointestinal/nutritional issues (difficulty chewing and swallowing, poor weight gain, gastroesophageal reflux, constipation), breathing dysrhythmias, sleep problems, seizures, and behavioral issues (anxiety, aggression, self-abusive behaviors), as well as others (eg teeth grinding). This list was discussed with caregivers of people with RTT associated with the International Rett Syndrome Foundation to provide input if any potential concerns were not included and a final list of choices was incorporated into the revised data collection forms. Caregivers also had the option to select “Other” and enter a free text description of the concern. The specified choices are shown in Table 1. The free text answers for the “Other” choices were reviewed manually. This manual review identified cases in which “Other” was chosen but the free text response clearly fell into the pre-specified choices (e.g., free text “hyperventilation” which fits into Rapid breathing or breath holding while awake). These were reclassified into the appropriate prespecified categories. The remaining free text responses were grouped into 13 additional clinical categories (e.g., “abdominal pain”, “gall bladder” and “vomiting” each contributed to the additional “Other GI” category), giving a final total of 34 distinct concern categories. The additional thirteen categories used for analyses are shown in Table 1.

### Participant information

From 2014 to March 2021, a total of 994 unique participants were enrolled and assessed, with the majority being participants with the diagnosis of Classic, or Typical RTT (Table 2). Participants were assessed longitudinally at pre-defined intervals based on age of enrollment, ranging from yearly to every other year. We excluded from the analyses participants with duplications of *FOXG1* (n=3), due to the small number of participants, and those grouped into the diagnostic category “Other”, which was genetically and clinically heterogeneous (e.g. people with *MECP2* mutations who do not meet clinical criteria for RTT; people with mutations in genes other than *MECP2, CDKL5, FOXGT, CDKL5* variants of unknown significance, unaffected siblings with *MECP2* duplications). Ultimately, we analyzed data on 925 participants, with n=641 having Classic RTT, n=84 having Atypical RTT, n=74 with *MECP2* Duplication Syndrome (MDS), n=67 with CDKL5 Deficiency Disorder (CDD), and n=59 having FOXG1 Syndrome (FS). The full breakdown of the participants, sex, and age groups is provided in Table 2. The *MECP2* mutation (or mutation groups) distributions for Classic and Atypical RTT is provided in Additional file 1: Table S1.

### Creation of weighted top concerns

To generate a list of top concerns, we analyzed these responses from the baseline visit for participants. To account for the relative importance of the concerns, we weighted each concern based on the rank order reported by the caregiver (weighted rank=1/Rank Order). Thus, for each participant, the First Concern received a weighted rank of 1, the Second Concern a weighted rank of 0.5 and Third Concern a weighted rank of 0.33 for each patient. The weighted scores for each category were summed for each diagnostic category, and for Classic RTT across age groups, severity groups, and mutation groups. A rank order for the top concern categories for each group was then created (top rank=higher weighted score), and the percentage for each concern category was calculated by dividing the weighted category score by the total of all weighted category scores for a given grouping that was analyzed. The group analysis was conducted on all participants for Classic RTT, as well as by age bins, severity, and *MECP2* mutations. Group analysis was also performed based on diagnostic categories (Classic RTT, Atypical RTT, MDS, CDD, and FS). Analysis by age bins, severity, and specific genetic mutation was only conducted for Classic RTT, as the overall number of individuals in the other disorders was limited when broken into further subgroups.

### Evaluation of weighted top concerns in Classic RTT

For Classic RTT, the weighted top concerns rankings were compared by calculating the 95% Confidence Interval (CI) for each concern using the standard deviation calculated from a binomial distribution. Significant differences in the proportion (presented as percentages) of concerns are reported at the p<0.05 level. Weighted top concerns rankings were organized by age, clinical severity, and common RTT-causing *MECP2* mutations [6, 7]. Clinical severity was assessed using two clinician-assessed measures, the Clinical Global Impression-Severity (CGI-S) and the RTT Clinical Severity Score (CSS) [28]. The CGI-S is a clinician assessment of overall clinical severity that is scored using a seven-point Likert score with one being normal function and seven being the worst level of function, and has established RTT specific anchors [28] which were systematically utilized in this study. The CSS is a clinical rating scale composed of 13 elements, each having Likert scores from 0-4 or 0-5 and a range of total scores from 0-58, with 0 again representing normal and 58 representing the most severe involvement [6].

### Comparison of top caregiver concern to clinician assessment of clinical features in Classic RTT

To evaluate the relationship of the First Caregiver Concern (not weighted) for an individual to clinical features noted by a physician, we compared the listed first concern to individual CSS item scores related to language, seizures, and hand use at the baseline visit. The percentage of caregivers who listed Lack of Effective Communication (Communication), Seizures, and Lack of hand use (Hand Use) for each item score for CSS Language, CSS Seizure, and CSS Hand Use score was calculated.

### Comparison of weighted top caregiver concerns across disorders

The weighted top concerns at the baseline visit for Classic RTT were compared to those for Atypical RTT, MDS, CDD, and FS. The smaller number of participants in these other diagnostic categories precluded further analysis by age. For Atypical RTT, we also developed weighted concerns for those regarded as being “Milder” or more “Severe” than Classic RTT, as people grouped into the Atypical RTT category have a bimodal severity distribution when assessed with the CSS [28]. Based on this, a cutoff of CSS<18 was used to define the “Mild” Atypical group and CSS>18 used to define the “Severe” Atypical group.

### Evaluation of top caregiver concern relative to caregiver impression of improvement/worsening

During study visits, caregivers were also asked to assess whether their child had improved, remained unchanged, or worsened over the last six months on a five-point Likert scale (Much Improved, Improved, Unchanged, Worse, and Much Worse), and indicate what was the main reason for their impression. In contrast to the top caregiver concern comparisons outlined above, for this analysis we did not restrict to only the baseline visit data but instead used the entire data set including repeated visits. We evaluated the responses for all visits for all participants by comparing the caregiver reasons for any Improvement (Much Improved or Improved) or any Worsening (Much Worse or Worse) and calculated the percentage of responses of caregiver reasons for Classic RTT, MDS, CDD, and FS. We also looked at percentage of Number 1 caregiver concerns (First Concern, unweighted) for each of these caregiver impression categories and disease categories.

## Results

### Top Caregiver Concerns in Classic RTT

**Figure 1:**
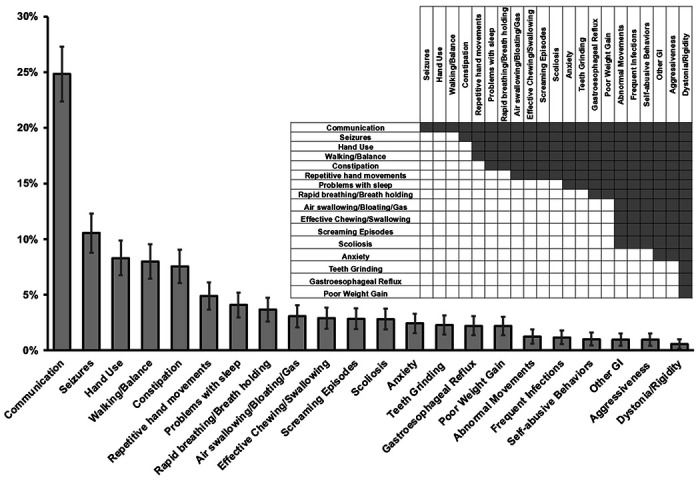
Weighted top caregiver concerns for Classic RTT. The percentage of each weighted top caregiver concern is presented with error bars representing the 95% CI. The inset presents the significant differences between weighted concerns as shaded cells (p<0.05).

The top five weighted concerns reported by caregivers for people with Classic RTT are 1) Lack of effective communication; 2) Seizures; 3) Lack of hand use; 4) Abnormal Walking/Balance; and 5) Constipation. Figure 1 displays the weighted concerns whose 95% C are above zero, with the pairwise differences shown in the inset of Figure 1. The full rank list of weighted concerns is presented in Additional file 2: Table S2.

Higher caregiver concern regarding the lack of key functional skills such as communication, hand use, and effective ambulation is not surprising for Classic RTT given the marked deficiencies affected individuals have in these functional domains; however, high level of caregiver concern regarding common clinical problems such as seizures and constipation is also notable. Importantly, caregivers rank “Lack of effective communication” as the top concern, with a weighted score far higher than the other concerns. This is very understandable as effective communication is fundamental to establishing any consistent interaction with the affected individuals.

### Variation in top caregiver concerns in Classic RTT between age groups

**Figure 2:**
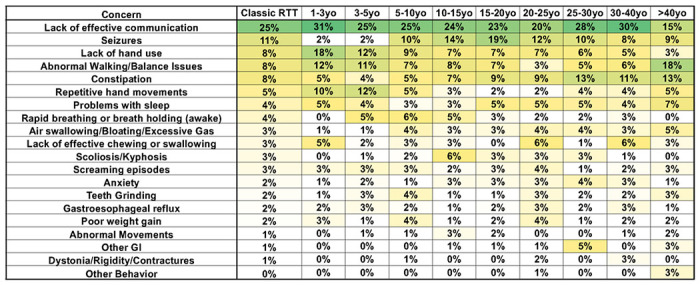
Weighted top caregiver concerns for Classic RTT varies by age. Top weighted concerns are listed on the left, with the order presented representing the rank order for all people with Classic RTT. Age bins are shown in subsequent columns. The heatmap color shows higher ranks (as indicated by the weighted percentage in the cells) as green, with intermediate ranks as yellow, and the lowest ranks in white. Only concerns with at least 3% in at least one cell are presented. Abbreviations: GI=gastrointestinal.

Viewing these concerns in Classic RTT according to the age of the individual provides a somewhat different picture (Fig. 2). Effective communication remains the top concerns until the oldest age group in which ambulation becomes paramount. Seizures, on the other hand are not a prominent concern until age 5, reaching a peak in the 15-20 year-old group and declining for the next four periods, consistent with the clinical observation of the peak period of seizure onset and severity [29]. Lack of hand use is the second highest concern until age 5, after which it declines steadily until it becomes much less important in the oldest group, despite the fact that hand function does not notably improve in the older age groups [30]. Constipation, a very common clinical problem [31], is a relatively steady concern throughout the first ten years, but then more than doubles in importance throughout the older age groups. Decreased mobility and ability to stand or walk individual could increase this issue. Rapid breathing or breath holding is non-existent as a concern until age 3, increases through age 15 and then declines to non-existent in the oldest group, following expected trends observed for the incidence of breathing abnormalities [32]. Concern regarding repetitive hand movements [30], is most significant during the first five years and then becomes less concerning for the remaining periods. Many of the less common concerns remain relatively constant with age, with notable exceptions that fit known age-dependent patterns of symptom onset and progression.

### Caregiver concerns in Classic RTT based on *MECP2* mutation

**Figure 3:**
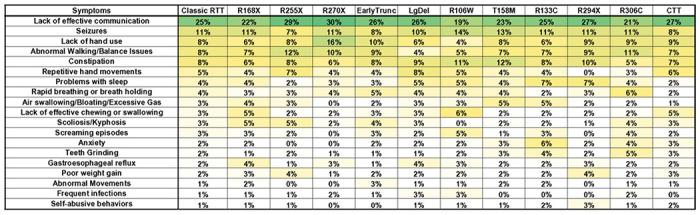
Weighted top caregiver concerns for Classic RTT across *MECP2* genotypes. Top weighted concerns are listed on the left, with the order presented representing the rank order for all people with Classic RTT. *MECP2* mutation groups are shown in subsequent columns, arranged with more severe mutations on the left. Heatmap color and abbreviations are as in Figure 2.

We compared variation in caregiver concerns for Classic RTT across the common, recurrent *MECP2* mutations (R168X, R255X, R270X, R106W, T158M, R133C, R294X, R306C) as well as mutation groupings that cause similar molecular disruption of the *MECP2* gene (Early Truncations, Large Deletions, C-terminal truncations [CTT]) compared to the combined concerns for people of all ages with Classic RTT (Figure 3). The overall pattern of top caregiver concerns was relatively consistent between mutation groups, although some differences were observed. Lack of effective communication remained the top concern across all mutation groups, although lack of hand use fell out of the top five concerns for R106W and R133C, gait problems for Large Deletions and R106W, and Constipation for R306C. In all these cases, these concerns remained within the top ten caregiver concerns, supporting the broad importance of these issues. Notably, while lack of effective chewing and swallowing ranked as the number 10 concern for all Classic RTT, for people with R106W this concern moved to the number 4 concern.

Broadly, the remaining top ten caregiver concerns remained consistent across the mutation groups, although some notable movement of specific concerns (as defined by a change of more than 50% of all Classic RTT percentages) was observed for some concerns. For example, repetitive hand movements were the number 6 concern for all Classic RTT but moved to number 19 concern for R294X. Similarly, air swallowing/bloating/excessive gas, the number 7 concern overall for all Classic RTT fell to number 20 for R270X and CTT. Notable movements into the top ten concerns include scoliosis/kyphosis for R168X, R255X and Early Truncations (all regarded as mutations associated with more severe clinical phenotypes), anxiety for R133C and R306C, and self-abuse for R294X. The increased rate of caregiver concerns for behavioral issues in mutations associated with less overall functional impairment (R133C, R294X, and R306C) is concordant with the clinically observed increased rates of behavioral problems in less severely affected individuals with Classic RTT [33].

### Caregiver concerns in Classic RTT vary by clinical severity

**Figure 4:**
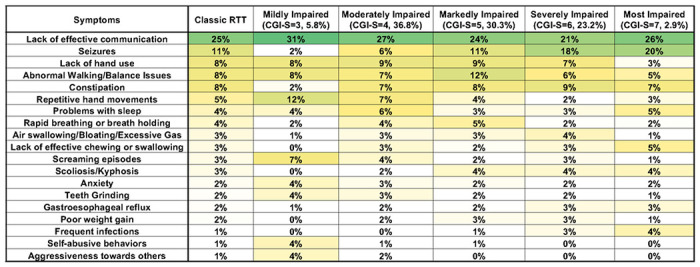
Weighted top caregiver concerns for Classic RTT across CGI-S scores. Top weighted concerns are listed on the left, with the order presented representing the rank order for all people with Classic RTT. CGI-S are shown in subsequent columns. Percentages of people in each CGI-S group is shown in the header. Heatmap color and abbreviations are as in Figure 2.

We evaluated top caregiver concerns in relation to clinician assessed severity by using the Clinical Global Impression – Severity (CGI-S) and RTT Clinical Severity Score (CSS). For the CGI-S, Lack of effective communication remained the top concerns across all severity groups (Figure 4). Within the CGI-S range that encompasses the bulk of the people with Classic RTT (CGI-S=4-6, accounting for 90.3%), the top concerns remain grossly similar, with some exceptions such as Repetitive hand movements dropping from number 6 to number 15 for the Severely Impaired group (CGI-S=6). Caregiver concern for seizures is very low in the Mildly Impaired group (CGI-S=3) and rises steadily with increasing CGI-S severity. Lack of hand use is a constant concern throughout the severity range until reaching the Most Impaired group (CGI-S=7), when it drops out the top 10 concerns. This is interesting as people within this severity group have the most overall impaired hand function, suggesting that caregivers shift focus to other clinical concerns when functional skills are severely impaired. This pattern is also seen for Abnormal walking/Balance issues, which are the number 2 concern for the Markedly Impaired group (CGI-S=5) but drop in the more severely affected groups (CGI-S=6-7), despite overall worsening gait function in people in these severity groups. While the Mildly Impaired (CGI-S=3) and Most Impaired (CGI-S=7) represent small fractions of the overall population (5.8% and 3.9%, respectively), there are interesting patterns of the top concerns. In the Mildly Impaired group, clinical features such as seizures, constipation, and chewing and swallowing drop, but behavioral concerns such as screaming episodes, anxiety, self-abusive behaviors, and aggressiveness increase to be within the top 10 concerns. This is in concordance with the observation that behavioral issues are more prominent in less severely affected individuals [33]. In the Most Impaired group, concerns for Frequent Infections, Gastroesophageal reflux, Other GI, and Other Health issues rise in concern.

**Figure 5:**
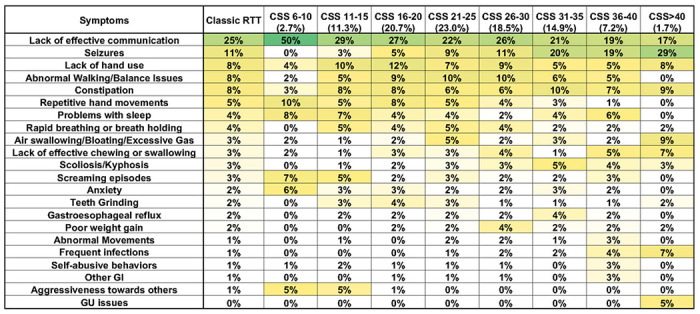
Weighted top caregiver concerns for Classic RTT across CSS scores. Top weighted concerns are listed on the left, with the order presented representing the rank order for all people with Classic RTT. CSS are shown in subsequent columns, arranged in goups from least to most severe. Percentages of people in each CSS group is show in the header. Heatmap color and abbreviations are as in Figure 2.

The comparison of concerns with CSS revealed a slightly different picture (Figure 5). Effective communication remained the top concern up to CSS of 36-40 where it was tied with seizures. At CSS >40 (most severe), effective communication concerns fell well below that for seizures. Seizures were not a concern at CSS 6-10 (least severe) but increased steadily from being relatively low in CSS 11-15 and 16-20 and then becoming increasingly prominent at higher scores. Walking/balance issues were modest at CSS 6-10 but increased through CSS 26-30 before falling dramatically at higher scores until not being a factor at CSS >40. Lack of hand use was of modest concern at CSS 6-10, was most prominent at CSS 11-15 and 16-20, and then declined thereafter. This pattern matched that seen using CGI-S as a measure of severity (especially since CSS and CGI-S are concordant [17]), with caregivers expressing less concern for functional hand use and gait for the most severely impaired individuals, despite these individuals being markedly impaired in these functional domains. In the most severely affected CSS groups, Frequent infections and GU issues concerns rise. Also as seen in the CGI-S analysis, behavioral concerns (Anxiety, Screaming episodes, Aggressiveness) are increased in the lowest severity groups and drop rapidly with increasing severity to being essentially nonexistent. Broadly summarizing the evaluation of top caregiver concerns, concern for problems with functional skills such as hand use and walking are most prominent in the middle severity groups (CGI-S=4-6, CSS=11-35); Seizures, GI problems, Scoliosis, and Frequent infection concerns becoming more important concerns in the most severely affected groups (CGI-S=7, CSS>36); and behavioral problems showing increased caregiver concern for the least affected groups (CGI-S=3, CSS<10).

To evaluate the relationship of the First Caregiver Concern (not weighted) for an individual to clinical features, we compared the Number 1 listed caregiver concern to individual CSS item scores related to language, seizures, and hand use (Table 3). The percentage of caregivers who listed Lack of Effective Communication (Communication), Seizures, and Lack of hand use (Hand Use) for each item score for CSS Language, CSS Seizure, and CSS Hand Use score is presented. Broadly, increasing severity in a domain on the CSS (higher score) is associated with increased percentage of that domain being ranked First concern, however this increase is not linear or completely consistent. Interestingly, while concern for Effective communication rises and peaks with a CSS Language score = 3 (vocalization, babbling), the percentage drops at the most severe CSS Language score = 4 (screaming, no utterances). A similar pattern is observed for the top concern Lack of hand use and the CSS Hand Use score, with the concern for hand use dropping at the highest CSS Hand Use score. These results are consistent with the finding that the caregiver concern for these functional skills declines in the highest severity groups of the global measures of severity, CGI-S and CSS.

Seizures are infrequently the top concern when seizures are absent (CSS Seizure = 0) and have the highest percentage as the top concern for the most severe CSS Seizure category (daily, intractable). An increase in seizures occurs as the top concern occurs for people with weekly seizures. A large percentage of caregivers of individuals without seizures (CSS Seizure = 0) ranked Lack of communication as the top concern (55.8%), which dropped rapidly as seizure burden increased. This pattern was also present for Lack of hand use, which dropped as the top concern rapidly as seizure burden increased. Overall, a pattern emerges that increasing seizure burden changes the frequency at which caregivers select the top concern, with decline in concern for functional skills and increase in seizures as the number one concern.

The top three weighted concerns for all Classic RTT is shown along the top, and the percentage of caregivers who indicated this being the first problem (Number 1 concern) for each of the CSS item scores is indicated in the cells. Percentages sum within column for each CSS item.

### Caregiver concerns in Atypical RTT

**Figure 6:**
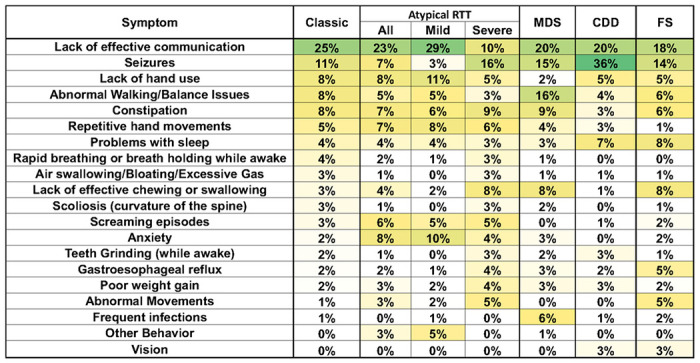
Weighted top caregiver concerns for Atypical RTT, MDS, CDD, and FS. Top weighted concerns are listed on the left, with the order presented representing the rank order for all people with Classic RTT. For Atypical RTT, total results for all people with Atypical RTT are shown, as well as those considered “Mild” (CSS<18) and those considered “Severe” (CSS>18). Heatmap color and abbreviations are as in Figure 2.

The top concerns for caregivers of individuals with Atypical RTT were generally similar to those reported in Classic RTT, with lack of effective communication being greatest and lack of hand use, walking/balance issues, and constipation each being less concerning, but similar (Figure 6). Seizures, however, were less concerning for parents or caregivers whose daughter was regarded as Atypical RTT. Based on the CSS scores, Atypical RTT has a binomial distribution above and below a CSS score of 18 [28]. When viewed as mild or severe Atypical RTT, several differences stand out. Among people with mild Atypical RTT, seizures were much less concerning, and lack of hand use and anxiety were of somewhat greater concern.

Behavioral concerns such as screaming, anxiety, and other behavioral problems were increased in mild Atypical RTT. For people with severe Atypical RTT, seizures became the top weighted concern, with lack of effective communication, walking/balance, and hand use dropping. Other features such as GI issues (gastroesophageal reflux, poor weight gain) become more prominent.

### Comparison of caregiver concerns between Classic RTT and other RTT-related disorders

Top concerns were compared across the different RTT-related disorders including MDS, CDD, and FS (Figure 6). Lack of effective communication remained the top weighted concern for both MDS and FS, but for CDD seizures become the top weighted concern, with more than 20% higher than for Classic RTT. This reiterates the known increase in overall seizure burden in people with CDD [34, 35]. Lack of hand use remained a significant concern in CDD and FS, but dropped markedly for MDS, but walking/balance concerns increased in MDS. Constipation was less of a concern for CDD, and repetitive hand movements was not a concern for FS. Notably, MDS has increased concern for chewing/swallowing and increased infections, the latter a noted problem in MDS [36–38]. Concerns about vision are present in people with CDD and FS, both of which have reported issues with cortical visual impairment [34, 39, 40].

### Caregiver impression of improvement

At each visit, caregivers provided a global impression of whether the overall condition of their child had improved, worsened, or remained unchanged. They were also asked to indicate the main reason for their overall global impression. The percentage of responses for each category for Classic RTT, MDS, CDD, and FS is provided in Table 4. For all the disorders, the number one reason listed for improvement was communication ability, with a wide range from 18% for FS to 37% for Classic RTT. The top caregiver reported reason for worsening in all disorders was seizures, ranging from 19% for Classic RTT to 63% for CDD. During visits in which the caregiver reported overall improvement, the first listed top concern for Classic RTT, MDS, and FS was lack of effective communication; however, for CDD the most frequent top concern was seizures (79%), although lack of effective communication in CDD remained the second most common concern when improvement was noted (33%). While there was relative commonality amongst the disorders regarding the most frequent first listed top concern in relation to caregiver impression of improvement or worsening, variation between the disorders was apparent for the second most frequently identified number one caregiver concern. For visits with caregiver noted improvement: Classic RTT, the next most frequently identified number one concern was hand use (10%); MDS, gait (17%), CDD, communication (33%); and FS, seizures (18%). In visits where the caregiver indicated clinical worsening: Classic RTT, communication (18%), MDS, tie between gait and frequent infections (both 9%), CDD, tie between communication, frequent infections, teeth grinding, and poor weight gain (all 5%); and for FS, lack of effective chewing and swallowing (22%). In conclusion, the main driver of improvement and worsening for all these disorders is communication ability and seizures (respectively) and the first caregiver listed concerns are similar between disorders; however, differences emerge between the disorders especially when less frequently observed first listed concerns are evaluated.

## Discussion

In this work, we analyzed the top caregiver concerns in people with RTT and Rett-related disorders from a large Natural history study to provide important information relevant to the design and selection of clinical outcome measures. The top concerns of caregivers of individuals with Classic RTT generally show that the functional skills lost in RTT (communication, walking, hand use) and clinical features common in RTT (seizures and constipation), are the most common concerns. Appropriately, the overall top weighted caregiver concern relates to their child’s inability to effectively communicate, which is not surprising as communication is fundamental to interpersonal connections and the dual loss of hand skills and spoken language profoundly impairs RTT individuals’ ability to effectively communicate. The presence of seizures and constipation in the top five weighted caregiver concerns for Classic RTT emphasizes the importance of these clinical problems in RTT. The pattern of top caregiver concerns in Classic RTT varies based on age, *MECP2* mutation, and clinical severity, with changes following expected patterns based on knowledge of how the relative prevalence of specific clinical issues changes with age or clinical severity, as observed in the natural history of RTT [29, 30, 32, 33]. For example, caregivers do not rank seizures as a higher concern until an age at which seizures become more prevalent in people with Classic RTT [29]. Interestingly, although impairments in functional skills such as hand use and ambulation are worse in more severely affected individuals and do not show marked improvements in older age individuals, the frequency at which caregivers rank these as higher concerns drops in the most severely affected groups and in older age groups. This could represent a caregiver shift from concerns related to impairment in functional ability to concerns regarding comorbid medical problems (e.g. seizures, scoliosis) that are, or become, more concerning overall in these groups. This also could reflect that caregivers for these older or more severely affected individuals have adjusted their expectations related to the long-standing functional impairments in these individuals and have developed a larger concern with other pressing medical issues. Our analysis of the relationship between top caregiver concerns and the assessed severity of specific features such as communication, gait, and seizures support this notion.

While broad similarities were identified in the top concerns of caregivers of people with Atypical RTT and Classic RTT, some differences were noted, especially when the Atypical RTT group was split into “milder” and “severe” groups, with a rise in the frequency of concerns related to behavior in the milder group and seizures in the severe group. This matches the pattern seen in Classic RTT when analyzed based on clinical severity and is consistent with the frequency at which these clinical issues are present in the respective groups.

The comparison of top caregiver concerns between RTT and other RTT-related disorders reinforces similarities between these disorders but also points out important differences. Communication remains the top concern across these disorders except for CDD, in which seizures become the overall top caregiver concern. This is concordant with the relative seizure burden and impact in CDD relative to the other disorders. Similarly, caregiver concern for frequent infections is only present at meaningful levels in people with MDS, reflecting the higher rate of infections in this population relative to the other disorders [36–38].

Obtaining information from individuals/caregivers about what matters most and is most impactful on daily life is now a priority and mandate from the FDA [23, 24] for the development of meaningful outcome measures selection for clinical trials. This is particularly important for the development of potentially disease-modifying interventions such as gene-based therapies as deciding what is considered changed should map to caregiver/individual concerns. Indeed, the FDA acknowledges that utilization of caregiver information may be needed for affected individuals with cognitive limitations [24]. Therefore, failure of a therapeutic intervention to modify these aspects of meaningful top concerns of a neurodevelopmental disorder may result in the lack of endorsement for effectiveness of a therapy from those responsible for providing the major care for these affected individuals. This issue will need to be addressed to determine the success of future trials. It stresses the need for outcome measures that can capture the breadth of disease impact across a heterogeneous range of severity, age and mutation. Due to the rarity of these diseases, robust outcome measures that address this challenge of heterogeneity are needed to achieve measurable outcomes in clinical trials. Across disorders, our analyses suggests that even if seizures were unchanged by a clinical intervention, an improvement in communication would be considered meaningful to caregivers.

While this work presents caregiver concerns captured from a large sample of caregivers of affected individuals, some limitations should be noted. The present study is based primarily upon cross-sectional data from the US NHS. The stability or change of these concerns over time for individuals is an important question, which will be a focus of future work utilizing the longitudinal data available from the US NHS. The majority of the data are derived from individuals with Classic or Atypical RTT; thus, the representation of these concerns among individuals with MDS, CDD, and FS is based on a much smaller group of individuals, suggesting that findings could vary with a larger sample of these rarer disorders. Additional efforts to gather information from those with these disorders could bolster these findings.

Another limitation is that caregivers were required to choose only the top 3 concerns and required to uniquely rank choices (ties or equivalence was not allowed). The consistency of the findings over such a large cohort and the fact that the major concerns align with our clinical experience and main symptoms in RTT, provides support that the data are an accurate representation of caregiver’s impression. Furthermore, caregivers were provided with 21 pre-specified concerns, with the option of choosing “other” and providing a free-text answer. While the pre-specified concerns were developed by review of clinical literature, expert clinical input, and RTT patient advocacy and caregiver input, it is possible that the range of items within the pre-specified concerns did not completely represent the range of possible concerns. This work is limited by the reliance on caregivers to identify concerns, rather than the affected individuals themselves. Unfortunately, the severe communication impairment in these disorders limits the ability for direct input from affected individuals. Finally, while caregivers’ concerns were identified, lacking was acquisition of caregiver impressions of the relative impact on the affected individual for the concern or ascertainment about the magnitude of change within a concern that would be meaningful. This represents an important avenue of future investigation that would further support the development and optimization of outcome measures for clinical trials in RTT and related disorders.

## Conclusion

The top concerns for individuals with RTT and RTT-related disorders are very similar across these different entities and are modified by age, clinical severity, and mutations as well as the specific diagnostic entity. The recognition of these caregiver concerns is critical in the development and selection of outcome measures for clinical trials in these disorders, as the measures should not be limited to a single domain and adequately assess top concerning features.

The understanding of what issues are most concerning and impactful for affected individuals should be a major consideration in the development and selection of outcome measures to be used in clinical trials, especially with the development of potentially disease-modifying interventions such as gene-based therapies. This work provides information on caregiver concerns for RTT and related disorders that can be used in this fashion, aligned with FDA recognition of the importance and guidance provided [23, 24], including the acknowledgement that utilization of caregiver information may be needed for affected individuals with cognitive limitations [24]. Parents and caregivers are likely to rate their impressions of improvement based on the symptoms they personally find most concerning. Failure to account for caregiver perceptions in these neurodevelopmental disorders may be viewed as a significant shortcoming by those responsible for providing the majority of care for these individuals. It is an issue that will require increased attention when assessing outcomes in future trials.

## Figures and Tables

**Table 1:** Top Concern Categories

Prespecified concern choices
Abnormal Movements (other than hand stereotypies)
Abnormal Walking/Balance issues
Aggressiveness towards others
Air Swallowing/Bloating/Excessive Gas
Anxiety
Constipation
Frequent infections
Gastro-esophageal reflux
Lack of effective chewing or swallowing
Lack of effective communication
Lack of hand use
Poor weight gain
Problems with Sleep
Rapid breathing or breath holding while awake
Repetitive hand movements
Scoliosis
Screaming episodes
Seizures
Self-abusive behavior
Teeth Grinding (while awake)
Vision
Other (please specify)

Created Terms
Attention/Cognition/Developmental Delay/ID
Drooling/Spitting
Dystonia/Rigidity/Contractures
Fatigue/Lethargy/Energy
GU issues
Hypotonia
None indicated
Other Autonomic
Other Behavior
Other GI
Other Health Issue
Other musculoskeletal
Respiratory/pulmonary

**Table 2:** Number of participants by diagnostic category and age bins.

Diagnosis	Total	Female	Male	Age (yrs)									
				<1	1 to 3	3 to 5	5 to 10	10 to 15	15 to 20	20 to 25	25 to 30	30 to 40	>40
**Classic RTT**	641	638	3	0	45	73	145	119	99	58	40	46	16
**Atypical RTT**	84	80	4	0	8	16	16	9	16	7	2	8	2
**MDS**	74	7	67	1	15	14	17	13	6	2	5	1	0
**CDD**	67	55	12	6	14	13	19	8	5	1	1	0	0
**FS**	59	35	24	6	16	8	20	6	2	0	0	1	0
**Total**	925	815	110	13	98	124	217	155	128	68	48	56	18

**Table 3:** Comparison of individual CSS item scores to number one caregiver concern.

	Number 1 concern		
CSS Language	Communication	Seizures	Hand Use
0 - Preserved, contextual	0.0%	0.0%	1.2%
1 - Short phrases only	0.9%	0.0%	1.2%
2 - Single words	11.5%	0.0%	6.2%
3 - Vocalization, babbling	61.9%	77.1%	50.6%
4 - Screaming, no utterances	25.7%	22.9%	40.7%
**CSS Seizures**	**Communication**	**Seizures**	**Hand Use**
0 - Absent	55.8%	1.2%	65.7%
1 - <Monthly	18.6%	12.3%	11.4%
2 - >Weekly to monthly	9.3%	12.3%	11.4%
3 - Weekly	6.6%	27.2%	5.7%
4 - More than weekly	4.4%	12.3%	2.9%
5 - Daily (intractable)	5.3%	34.6%	2.9%
**CSS Hand Use**	**Communication**	**Seizures**	**Hand Use**
0 - Conserved	12.8%	3.7%	11.4%
1 - Acquired on time, partially conserved	16.4%	8.6%	20.0%
2 - Acquired late, partially conserved	11.1%	8.6%	2.9%
3 - Acquired and lost	53.1%	69.1%	57.1 %
4 - Never acquired	6.6%	9.9%	8.6%

**Table 4:** Caregiver Impression of Improvement

Caregiver Impression		Classic RTT	MDS	CDD	FS
**Unchanged**		**52%**	**33%**	**38%**	**43%**
**Improved**		**27%**	**47%**	**49%**	**44%**
Top Caregiver Reason	Communication	37%	30%	29%	18%
First Concern	Communication	30%	42%	33%	27%
First Concern	Seizures	8%	10%	47%	18%
**Worsening**		**21%**	**20%**	**13%**	**13%**
Top Caregiver Reason	Seizures	19%	42%	63%	22%
First Concern	Seizures	21%	55%	79%	39%
First Concern	Communication	18%	3%	5%	6%

## Data Availability

The datasets from the Rett syndrome and Rett-related Disorders Natural History Study (NHS) have been deposited to the database of Genotypes and Phenotypes (dbGAP) repository, phs000574.v1.p1 and hyperlink to dataset(s) in https://www.ncbi.nlm.nih.gov/projects/gap/cgi-bin/study.cgi? study_id=phs000574.v1.p1, and are deposited to dbGAP per a predefined schedule at regular intervals. Additionally, datasets used for the analysis conducted within this work are available from the corresponding author on reasonable request and pursuant to any required data transfer and use agreements.
